# Armored polymer-fluid gels with integrated damping and impact protection across broad temperatures

**DOI:** 10.1126/sciadv.adv5292

**Published:** 2025-04-09

**Authors:** Guoqing Chen, Jiabin Wu, Zhenwu Wang, He Zhu, Shiping Zhu, Qi Zhang

**Affiliations:** School of Science and Engineering, The Chinese University of Hong Kong, Shenzhen, Shenzhen 518172, P. R. China.

## Abstract

Unpreferable vibrations and impacts pose substantial risks to sensitive devices, structures, and the human body, demanding materials capable of providing both high energy dissipation and impact protection across a broad temperature range. Traditional damping materials often fail to meet these demands because of a trade-off between damping and mechanical strength. We introduce an innovative strategy to fabricate armored polymer-fluid gels (APFGs) that combine high damping and high modulus for effective damping and impact protection under extreme conditions. By using a controlled surface cross-linking process through diffusion, we greatly enhance the mechanical strength of polymer-fluid gels without sacrificing their damping capabilities. This asymmetric design results in an unprecedented loss factor (tanδ > 0.5 from −45 degrees to 135 degrees Celsius, peaking at tanδ = 2.2) while achieving a tensile modulus of 20 megapascals. This method resolves the long-standing damping-modulus trade-off, positioning APFGs as promising candidates for robust damping and impact protection in electronics and human motion applications.

## Teaser

An innovative approach is proposed for the design and fabrication of anisotropic armor-core-structured polymer-fluid gels.

## INTRODUCTION

Unavoidable disturbances, such as vibration and impact, can cause minor or severe mechanical damage, greatly shorten the lifespan of objects, generate noise during electronic operation, and sometimes even cause harm to the human body (*1*–*3*). Damping materials play crucial roles in converting these disturbance-induced mechanical energies into heat (*4*), thereby mitigating vibrations and minimizing impacts in various applications such as construction (*5*), transportation (*6*–*8*), and electronics (*9*–*11*). Mechanical disturbances often occur across a wide temperature range, sometimes under extreme conditions, where precision instruments become more fragile at low temperatures, making protection particularly critical. Traditional damping materials, mainly based on polymers, rely on viscoelasticity closely tied to their glass transition region (*12*–*14*). However, at low temperatures (<−20°C), their damping performance largely declines as polymers enter a brittle glassy state, restricting chain flexibility (*15*–*17*). While low-glass-transition-temperature (*T*_g_) polymers offer a solution for cold environments, they underperform under moderate conditions (*18*–*20*). Thus, creating materials that provide efficient damping over a broad temperature spectrum while maintaining high mechanical strength for impact protection has been a longstanding challenge.

In addition to vibrations, transient impacts are omnipresent and require high-modulus materials to minimize excessive deformation and prevent material failures (*21*, *22*). However, a longstanding trade-off exists between damping and modulus: Increasing the modulus through chain cross-linking or incorporating rigid fillers typically restricts chain segmental motion and decreases internal friction, ultimately diminishing damping effectiveness (*23*). Conventional approaches attempt to address this challenge by composite or anisotropic design, but they tend to be complex, poorly integrated, and limited by poor temperature stability (*24*–*26*). To address these challenges, we propose an effective approach to combine broad-temperature damping and high modulus by armor-core-structured gels. The gels integrate polymer fluids into polymer networks, forming polymer-fluid gels (PFGs) for effective damping across an ultrawide temperature range. They leverage the fluid phase to enhance flexibility and energy dissipation and the polymer network to provide mechanical stability (*27*). By precisely controlling cross-linking in a thin surface layer, we introduce the anisotropic armor-core structure that imparts high modulus to the material (*28*), enabling simultaneous achievement of both excellent damping and high modulus. The gradient and integrated structure offers distinct advantages over traditional complex composite systems, such as constrained damping materials (*29*).

We demonstrate the effectiveness of this approach by copolymerizing acrylic acid (AA) and hydroxyethyl methacrylate (HEMA) within polyethylene glycol (PEG) fluid, followed by surface cross-linking in Fe^3+^ solutions to form an armor-core structure (*30*). The resultant armored PFG (APFG) exhibits a high loss factor (tanδ > 0.5 from −45° to 135°C), with a substantially increased tensile modulus (from 0.2 to 20 MPa) and puncture resistance compared to PFGs. The material effectively dissipates energy, reduces rebound by more than 98%, and effectively lowers vibrational forces by up to 72% at −20°C. Furthermore, the APFG extends the operational lifetime of electronic devices, ensures their stable performance, and offers superior protection against transient impacts, safeguarding both electronics and human joints. This work introduces a breakthrough solution to the long-standing damping-modulus dilemma, positioning these materials as promising candidates for various applications in vibration damping and impact protection, particularly in extreme thermal environments.

## RESULTS

### Concept of APFGs

**Fig. 1.**
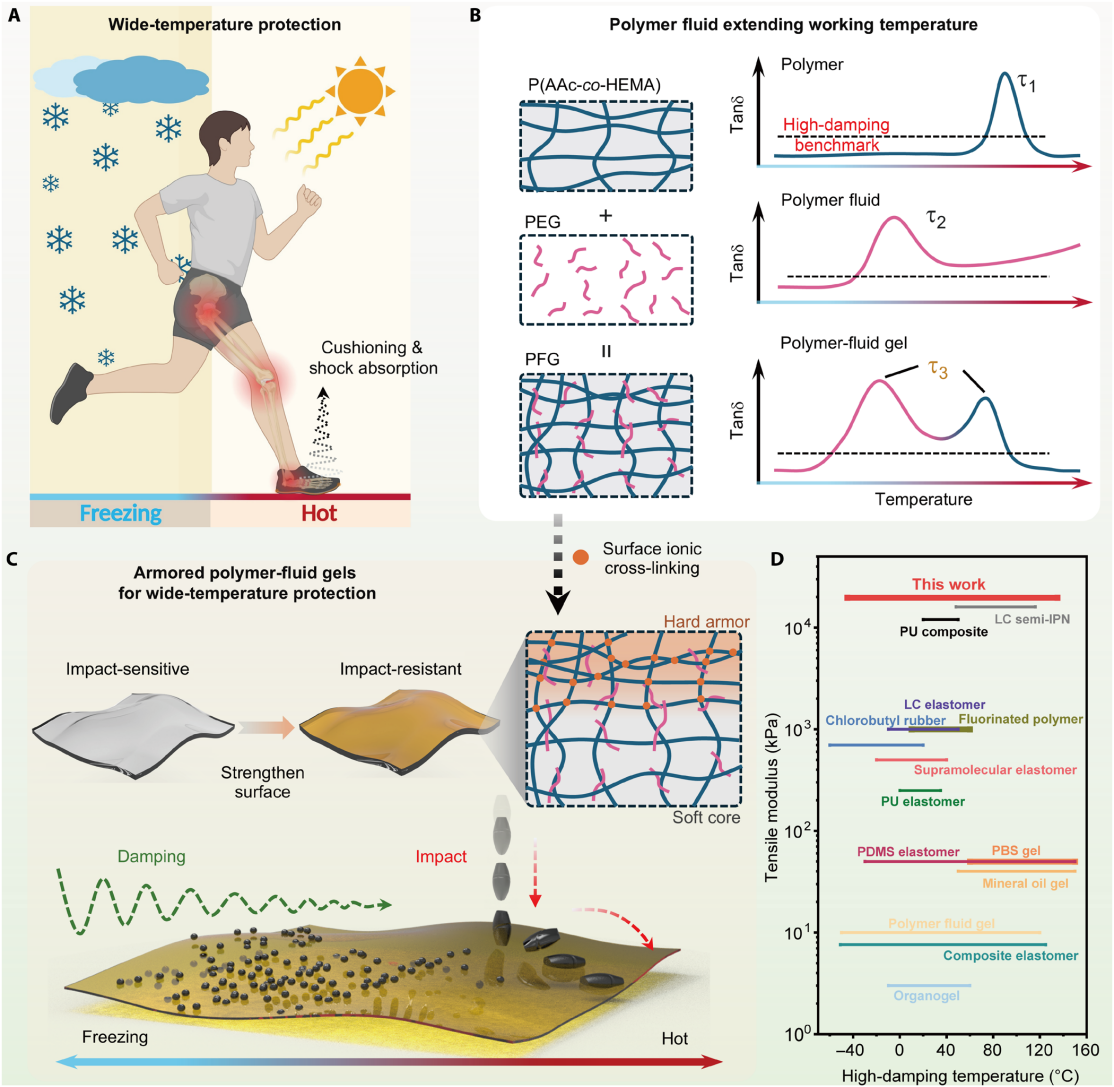
Design strategy, fabrication process, and performance of APFGs. (**A**) Daily scenario that requires broad-temperature-range protections by damping materials. (**B**) Design principle of wide-temperature-range damping PFGs by incorporating different chain movement modes and relaxation times of the polymer fluid and network. (**C**) Fabrication of APFGs from PFGs through a one-step soaking strategy and for its application in impact protection. (**D**) Comparison of reported materials displaying their moduli and applicable damping temperature ranges. Detailed data can be found in table S1.

Damping materials play essential roles in cushioning and shock absorption applications. Given that most elastomers exhibit limited loss factors (typically < 0.1), materials recognized with a high damping ability usually have a tanδ > 0.5 (*10*, *18*). Because of intrinsic viscoelastic properties, current damping polymer materials only function well in a narrow temperature range, usually at room temperature (RT). For example, the soles of sports shoes, made of polymers like rubbers or polyurethanes, can reduce impacts and absorb shocks effectively to protect human articulations and provide better comfort. However, their damping performance is limited and can be extensively suppressed at low or high environmental temperatures because of the limited loss factors and narrow dissipative regions, sacrificing comfort and health care efficacy (Fig. 1A). Such dilemmas also arise frequently in household compliance and industry applications. Achieving a broad, effective damping temperature range (tanδ > 0.5 over a temperature interval broader than 100 K) remains an important challenge.

Gels with polymer fluids as solvents are increasingly used in damping materials because of their stability, processability, and adjustable mechanical properties (*31*). By regulating the relaxation time of both solvent and polymer matrix, we can finely tune the damping performance to achieve wide-temperature damping (*20*). As a representative in this study, we chose AA and HEMA monomers copolymerized in a low-molecular-weight PEG solvent to create the PFGs. On the one hand, for the copolymer network, damping behavior relies on segmental chain movements, demonstrating a narrow damping range associated with a high relaxation time τ_1_. On the other hand, linear PEG introduces chain reptation movements that facilitate the relaxation process, leading to a lower relaxation time τ_2_ and broader damping range, while only liquid-state PEG showed no practicality. Fortunately, this combination yields homogeneous gels with a relaxation time τ_3_ and distinct peaks at separated temperatures (i.e., frequencies), enabling high loss factors across a wide temperature range, because of excellent compatibility between copolymer networks and PEG (Fig. 1B) (*18*).

In addition, high modulus and impact resistance are also essential for wide-temperature protection, as they ensure durable and stable functionality. Nevertheless, achieving wide-temperature-range damping by PFGs intrinsically compromises the material’s *T*_g_ and these mechanical properties, making them sensitive to impacts. To address these issues, we introduced surface cross-linking to enhance mechanical performance by immersing the PFGs in a Fe^3+^ solution prepared by dissolving FeCl_3_·6H_2_O in PEG. The diffusion of ferric ions into the gel forms strong ionic physical cross-links between Fe^3+^ ions and ionized COO^−^ groups of AA (Fig. 1C) (*32*). This armored layer enhances the material’s mechanical properties without substantially affecting the damping performance of the softer substrate. As a result, the APFGs exhibit excellent damping capabilities over a wide temperature range, along with enhanced impact protection for diverse applications (Fig. 1C). Overall, by precisely tailoring molecular design and controlling the ionic cross-linking, APFGs achieved an ultrawide working temperature range with high loss factors (tanδ > 0.5 from −45° to 135°C) and a high tensile modulus (20 MPa), a challenging combination to attain in homogeneous polymers. This performance surpasses most reported damping materials across multiple categories, including elastomers (*33*–*39*), gels (*18*, *40*–*42*), and polymer composites (Fig. 1D and table S1) (*19*, *43*).

### Preparation and characterizations of APFGs

We first conducted detailed investigations into the design of PFGs for optimal performance. Unlike other small-molecule solvents that typically reduce chain friction in conventional hydrogels or organogels (*44*), PEG forms dense sacrificial hydrogen bonds with both AA and HEMA, ensuring high energy dissipation through reptation motions (*45*). Moreover, PEG also demonstrates superior properties including antifreezing and nonvolatility compared to water in hydrogels. A series of PFGs were first synthesized by copolymerizing AA and HEMA in the presence of PEG solvent in a weight ratio of 1:1 for monomers and PEG. We obtained the Fourier transform infrared (FTIR) spectra of homopolymer PAA, PHEMA, PEG 400, and the corresponding PFG-400 gel (fig. S1). The shifts of characteristic peaks of carboxyl and hydroxyl groups in the PFGs compared to each component to higher wavenumbers indicate the formation of intermolecular hydrogen bonds between the copolymer and PEG (*46*). This helps to plasticize the copolymer network, which varies distinctively on the basis of PEG’s molecular weight. To confirm this, we synthesized four different PFGs containing PEG 200, 400, 600, and 800, all demonstrating a homogeneous phase with similar structures. They demonstrated corresponding changes of the *T*_g_ and loss factor, as revealed by dynamic mechanical analysis (DMA) (fig. S2, A to C). The monomer ratio between AA and HEMA also altered the loss factor peak value and peak temperature because of stronger interactions between PEG and AA compared to HEMA (fig. S2D). However, the hydroxyl group on HEMA monomers facilitates self-condensation reactions to form self-cross-links (*47*), which are crucial for maintaining gel stability because gels composed solely of PAA and PEG tend to melt at elevated temperatures (fig. S3). On the basis of the optimizations, we selected PFG-400, with a weight ratio of 1:1:2 for AA, HEMA, and PEG 400, as the representative material for subsequent analysis, as it provides balanced *T*_g_ and loss factor over a broad applicable damping temperature range. As shown in fig. S2E, temperature-dependent DMA from −50° to 150°C reveals two separate characteristic peaks ascribed to dominant segmental motions at lower temperatures and chain reptations at higher temperatures, which supports our design presented in Fig. 1B. This design can thus broaden the effective damping temperature range to −45° to 135°C. The self-cross-linking of HEMA limited excessive relaxation of the polymer network, resulting in only a mild decrease in storage modulus (*G*′) and loss modulus (*G*″) at elevated temperatures (fig. S2F).

**Fig. 2.**
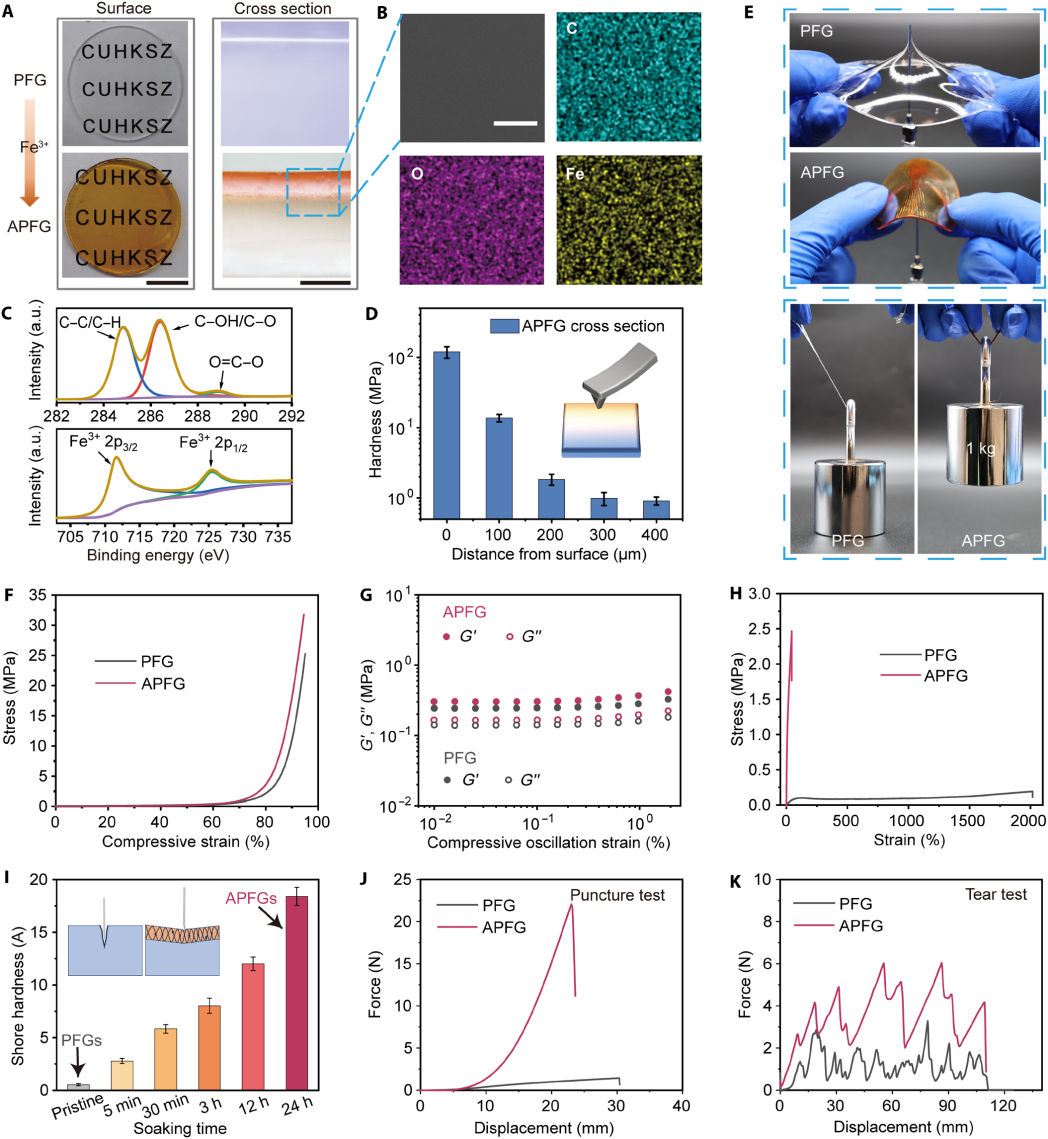
Characterizations and mechanical properties of armored PFGs (APFGs). (**A**) Photographs of PFGs and APFGs showing surface morphology (scale bar, 1 cm) and cross-sectional structure (scale bar, 200 μm). (**B**) Cross-sectional SEM images (scale bar, 50 μm) of APFGs, supplemented by energy-dispersive x-ray spectroscopy mapping of carbon (C), oxygen (O), and iron (Fe). (**C**) XPS spectra of APFGs showing the C 1s and Fe 2p binding states, confirming successful ionic cross-linking and the formation of surface complexes. a.u., arbitrary units. (**D**) Cross-sectional hardness gradient of APFGs, indicating a diffusion-hardened surface transitioning into a softer internal region (sample size, *n* = 5 each). (**E**) Mechanical performance differences between PFGs and APFGs demonstrated through puncture tests of 1-mm-thick films by a needle and weightlifting tests with a 1-kg load. (**F**) Compressive stress-strain curves comparing PFGs and APFGs. (**G**) Compressive DMA test for PFGs and APFGs. (**H**) Tensile stress-strain curves of PFGs and APFGs. (**I**) Shore hardness evolution of PFGs with increasing immersion time in Fe^3+^ solution (*n* = 5 each). h, hours. (**J**) Force-displacement curves during puncture tests comparing PFGs and APFGs. (**K**) Tear resistance force-displacement curves for PFGs and APFGs.

Detailed experiments were conducted to investigate physiochemical property changes from PFG to APFG. As cross-linking occurred in the surface layer of the gel, the material successfully transformed into APFG after soaking for 24 hours. Unlike uniformly cross-linked polymers, the surface modulus and impact resistance of the resultant APFGs can be greatly enhanced while maintaining the polymer composition and high damping of the inner core. After a 24-hour soaking period, the originally transparent PFG gradually turned reddish-yellow, as shown in Fig. 2A and fig. S4A. The transparency was reduced because of the absorption of ultraviolet (UV)-visible light by Fe^3+^ (fig. S5) (*48*). The cross-linked layers with an apparent reddish-yellow color could be directly observed by optical microscopes from a cross-sectional view, where the color gradation indicated the degree of cross-linking (Fig. 2A and fig. S4B). Thus, the thickness of the cross-linked layers at different soaking times was roughly estimated, showing an increase to about 160 μm after 24 hours of soaking (fig. S6). Because of the mild and nondestructive nature of the cross-linking process through diffusion, the material remained unseparated from its shell to the core, as revealed by scanning electron microscopy (SEM). Elemental mapping via energy dispersive spectroscopy also confirmed a substantial distribution of Fe in the cross-linked area compared to the pristine PFGs (Fig. 2B and fig. S7). Other surface chemical characterizations, including x-ray photoelectron spectroscopy (XPS) and FTIR, were conducted to verify the successful cross-linking by Fe^3+^. Figure 2C and fig. S8 show the XPS results of both PFGs and APFGs, and emerged peaks at 711.7 and 725.5 eV confirmed the presence of ferric ions in the APFG surface (*49*). The FTIR characteristic peak of carbonyl groups of AA also shifted from 1724 to 1702 cm^−1^ in a soaking time–dependent manner, corresponding to the ionic interaction between AA and Fe^3+^ (fig. S9). After confirming the successful doping of ferric ions, we investigated its effects on the APFG’s hardness by nanoindentation, as depicted in Fig. 2D. The hardness gradient along the diffusion direction was calculated from load-displacement curves in fig. S10 using the Oliver-Pharr method (*50*). The hardness at the armored outmost shell reached 119.7 MPa and gradually decreased to 13.8, 1.8, 1.0, and 0.9 MPa as the indented position moved 100 μm each time to the soft core. This gradient change highlights the enhancement in material hardness because of cross-linking and confirmed the presence of an ion concentration profile at the surface of APFGs, mainly governed by a diffusion process. The diffusion can eventually penetrate the gel as soaking time increases.

### Mechanical properties of APFGs

In a macroscopic view, the PFG showed limited mechanical performance with respect to the modulus and strength because of the high plasticization from a large proportion of liquid PEG 400 within the polymer network. The PFG at RT exhibited a soft state and was incapable of maintaining a stable shape under gravity. Conversely, the formation of hard armor through surface cross-linking effectively enhanced the shape retention ability of PFGs (fig. S11). Moreover, this hard armor substantially improved the puncture resistance and stress tolerance in APFGs as the hardness and ultimate strength increased. As shown in Fig. 2E and movie S1, a 1-mm–thick PFG film was easily penetrated by a needle, whereas the APFG film resisted the puncture. Similarly, when a 1-kg weight was lifted using both specimens, the PFG strip elongated until the break, whereas the APFG strip securely lifted the weight without damage. Overall, the ionic cross-linking treatment for mechanical improvement via ferric ion diffusion is effective, convenient, nondestructive, and highly adaptable. By combining this strategy with the material design of PFGs, it is expected to successfully achieve the armor-core structure and have the damping-protection function.

To quantify the changes in mechanical properties, we conducted various characterizations on both PFGs and APFGs. The continuous armored structure of APFGs exhibited anisotropic mechanical behavior in response to external stimuli. In compression tests using specimens with a thickness of 5 mm and a diameter of 15 mm, both the pristine PFGs and APFGs showed strain-increasing moduli, reaching high strengths of 25 and 32 MPa at 95% strain, respectively (Fig. 2F). This demonstrates their capability to withstand high stress without rupture, outperforming many conventional gels (table S2) (*18*, *51*–*55*). The minimal changes in modulus and strength can be attributed to the relatively minor contribution of the thin hard layer in the compressive direction, with the major contribution coming from the soft inner core. Compressive DMA tests corroborated these findings, showing minimal changes in *G*′ and *G*″, thereby preserving damping performance (Fig. 2G). However, in uniaxial tensile tests, the presence of hard armor greatly altered stress-strain curves because of confinement and reinforcement effects (Fig. 2H). Non–cross-linked pristine gels could stretch up to an extraordinary 2000% strain, with an ultimate stress of only 0.29 MPa. After surface ionic cross-linking, the maximum elongation was reduced to about 50%, while the strength increased to more than 2.5 MPa.

The gradient changes in mechanical properties can be controlled by varying the surface treatment conditions, such as the soaking time of PFGs or the concentration of Fe^3+^ solutions. As shown in fig. S12, the compressive modulus of the gels increased gradually from 0.18 to 0.26 MPa as the soaking time extended from 5 min to 24 hours. In contrast, the tensile modulus markedly increased from 0.08 to 20.04 MPa, a remarkable 250-fold enhancement. Yielding and necking were also observed in tensile tests, which further confirmed the formation of armor-core structures (fig. S12D). Similar enhancements were observed by varying Fe^3+^ solution concentrations, with increased ion concentration resulting in improvements in modulus and Shore hardness comparable to extended soaking time (fig. S13).

Being stiffer than the inner core, the armors also improved the gels’ resistance to external indentation, a critical factor for applications in nonconstrained damping scenarios where transient impacts could damage materials with weak external resistance (*56*). Shore durometer tests showed an increase in hardness from about 0.5 to more than 18.4 A after 24 hours of immersion, ensuring durability against impacts (Fig. 2I). As a result, the armor conferred improved resistance to punctures and tears. The maximum puncture force of the 1-mm-thick APFG was measured at 22.1 N, representing a 15-fold increase over the 1.4-N puncture force of PFG (Fig. 2J). In addition, tear resistance was markedly enhanced, with the average tear force tripling from PFG to APFG (Fig. 2K). The puncture energy increased from 0.02 to 0.14 J, while tear energy rose from 127.2 to 371.9 kJ m^−2^. Overall, the combination of damage resistance and effective damping allows the APFGs to dissipate energy effectively, minimizing permanent damage upon impact.

### Damping performance of APFGs

**Fig. 3.**
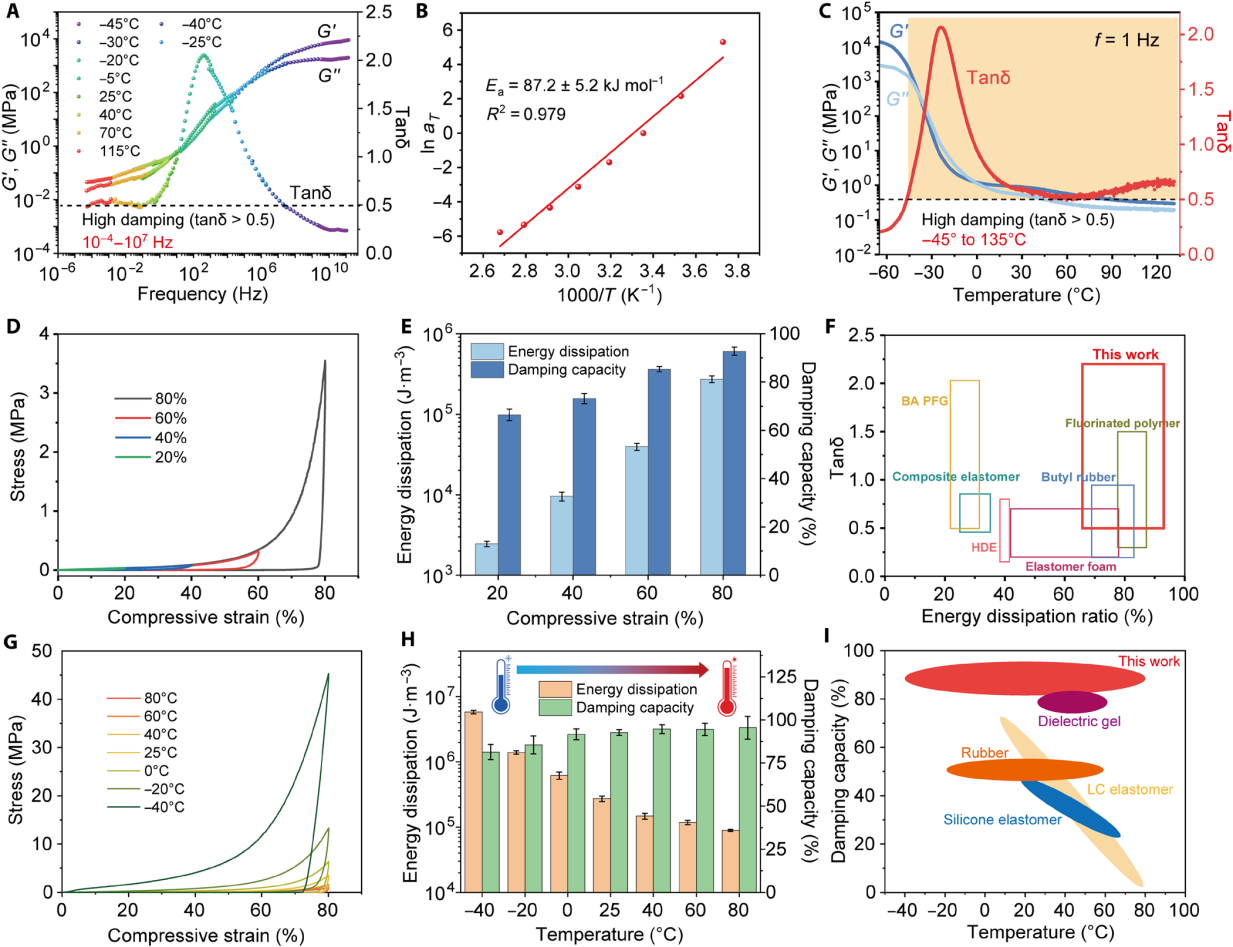
Damping and energy dissipation properties of armored PFGs (APFGs). (**A**), Master curve derived from TTS analysis with a reference temperature of 25°C, illustrating the frequency-dependent damping characteristics of APFGs. (**B**) Calculation of relaxation activation energy using linear fitting of logarithmic of horizontal shift factors (ln*a*_*T*_) against reciprocal temperatures (1/*T*) based on the Arrhenius equation. (**C**) Temperature-dependent DMA results of APFGs, highlighting efficient damping (loss factor >0.5) from −45° to 135°C. (**D**) Cyclic compressive stress-strain curves of APFGs under varied strains. (**E**) Energy dissipation and damping capacity of APFGs obtained from the loading-unloading curves at varied strains (*n* = 3 each). (**F**) Comparative diagram showcasing the loss factor range and energy dissipation ratios of APFGs in relation to other reported materials. (**G**) Cyclic compressive stress-strain curves of APFGs under varying temperatures. (**H**) Energy dissipation and damping capacity of APFGs as a function of temperature obtained from the loading-unloading curves (*n* = 3 each). (**I**) Ashby plot comparing energy dissipation ratios and damping capacity of APFGs with other materials across varying temperature conditions.

To delve into the damping performance of APFGs, we conducted compressive DMA tests to obtain critical figures of merit, including *G*′, *G*″, and tanδ. Strain-dependent tests were first carried out from 0.01 to 2% compressive strain at a fixed frequency of 1 Hz and RT to identify the linear region of the samples, in which cylindrical samples featured cross-linked armors on their top and bottom surfaces. The results in fig. S14 showed that introducing the armored structure through appropriate treatment minimally affects the damping ability in compressive mode, as specimens with various cross-linking times and thickness ratios exhibit similar *G*′, *G*″, and tanδ values. This suggests that the optimal performance for specific applications can be finely tailored. Multifrequency tests at separate temperatures were conducted, and time-temperature superposition (TTS) analysis was performed to obtain the master curve and investigate the damping behavior at extreme frequencies. Both *G*′ and *G*″ exhibited a continuous increase, and the highly damping range expanded from 10^−4^ to 10^7^ Hz (Fig. 3A). The activation energy of relaxation calculated was 87.2 kJ mol^−1^, much smaller than that of traditional damping elastomers, indicating the high mobility of the polymer chains (Fig. 3B). Furthermore, APFGs exhibited an ultrawide damping temperature range spanning from −45° to 135°C, with all loss factor values exceeding 0.5 and a prominent peak of 2.2 appearing at −25°C, around the glass transition region where maximum dissipation occurs (Fig. 3C). Notably, despite the high moduli at low temperatures, APFGs showed negligible modulus loss as the temperature increased from RT to 135°C, distinguishing them from other linear polymer gels (Fig. 3C). As shown in Fig. 1D, the usually irreconcilable high modulus and broad damping were achieved by our design, which demonstrated distinct superiority compared to existing materials.

In practical applications of damping materials, energy dissipation is another critical factor. Typically, cross-linked elastomers store a substantial portion of energy within their elastic network during deformation, which can be recovered through reverse motions with low hysteresis. To evaluate this capability in APFGs, we generated compressive load-unloading curves at 20, 40, 60, and 80% strains. The engineering stress values increased rapidly with rising strains during loading, resulting in effective energy absorption from external sources (Fig. 3D). The hysteresis loop also expanded with increased compression, correlating with dissipation ratios. As summarized in Fig. 3E, the dissipation amount rose markedly from 2.5 kJ m^−3^ at 20% strain to 9.6, 39.6, and 272.7 kJ m^−3^ at subsequent 40, 60, and 80% strains, respectively. The damping capacity (energy dissipation ratio) also showed strain dependency, increasing from 66 to 93% across these strains, surpassing most reported values. This superiority is further evidenced when comparing APFGs with other damping materials in terms of energy dissipation ratio and tanδ range (Fig. 3F), highlighting exceptional performance compared to the existing literature (*18*, *19*, *36*, *38*, *57*, *58*).

Temperature-dependent cyclic compressions at 80% strain were also carried out from −40° to 80°C to evaluate energy dissipation under extreme temperatures (Fig. 3G). The moduli of the material were highly temperature dependent, with energy dissipation peaking at lower temperatures (5795.7 kJ m^−3^ at −40°C). Conversely, the dissipation ratio slightly increased from 81.4% at −40°C to 95.5% at 80°C (Fig. 3H). The substantial energy dissipation across wide temperature ranges outperforms many reported materials, which often struggle with temperature sensitivity in energy dissipation (Fig. 3I) (*10*, *59*–*61*).

### Wide-temperature-range damping of APFGs

**Fig. 4.**
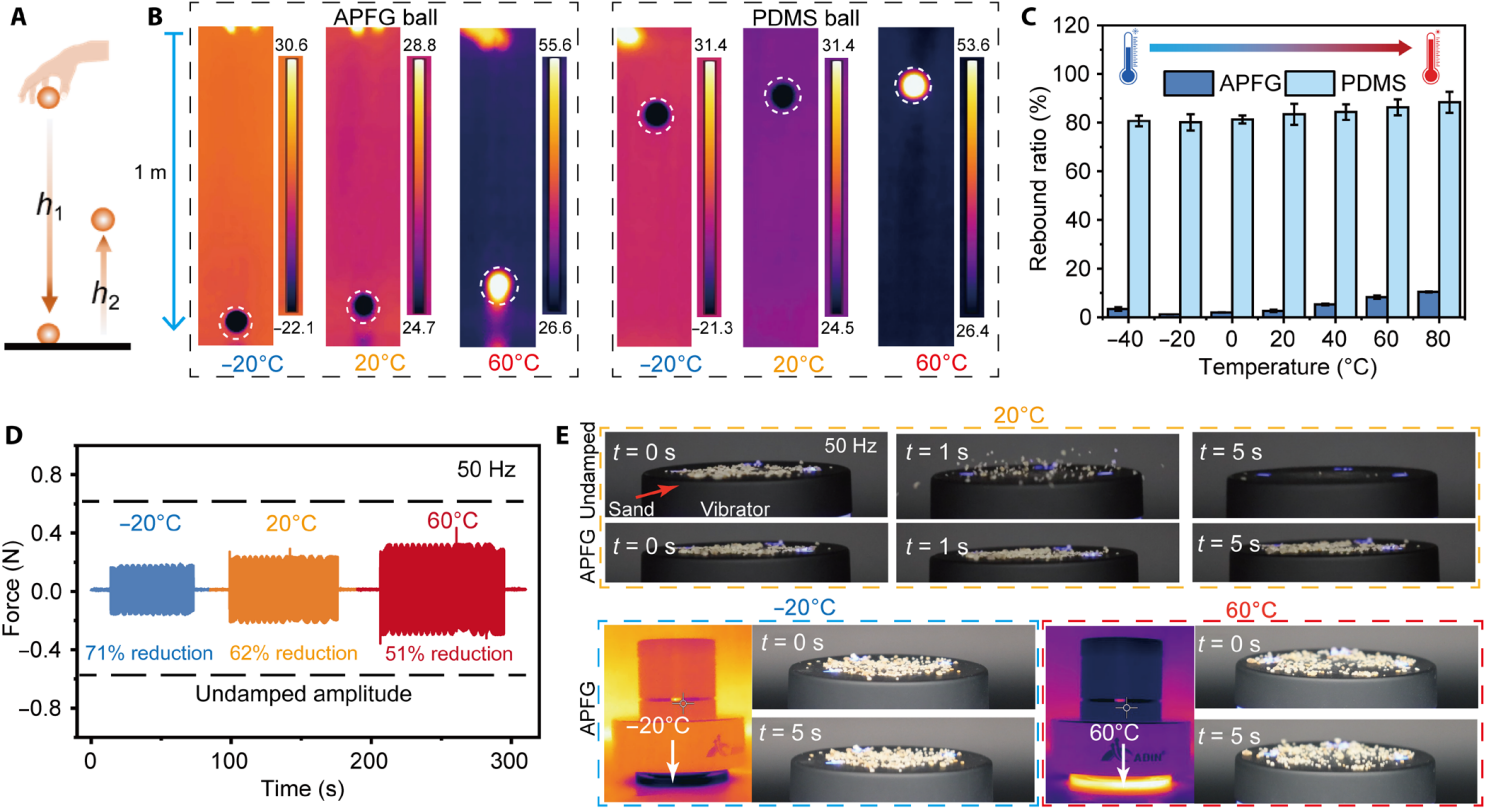
Energy dissipation and vibration damping performance of armored PFG (APFG) across wide temperature ranges. Energy dissipation in wide temperatures: (**A**) Schematic illustration of rebound tests. (**B**) Infrared thermal images showing maximum rebound heights of APFG and PDMS balls dropped from a fixed height of 1 m at −20°, 20°, and 60°C. (**C**) Calculated rebound ratios of APFG and PDMS balls across temperatures ranging from −40° to 80°C (*n* = 5 each). Vibration damping in wide temperatures: (**D**) Temperature-dependent vibrational force damping performance of APFG dampers under 50-Hz vibration, showing reductions in force amplitude at −20°, 20°, and 60°C. (**E**) Demonstrations of a 5-mm-thick APFG damper preventing sand grains from vibrating at −20°, 20°, and 60°C

Given their robust damping performance, APFGs are well suited for applications involving energy absorption and the attenuation of vibrational forces. To demonstrate the energy dissipation capabilities of APFG dampers, we conducted rebound tests where a ball was released from a specified height (Fig. 4A). The rebound ratio, denoting the ratio of rebound height to release height, indicates the material’s macroscopic damping and energy dissipation ability. In our temperature-varied rebound tests, both an APFG and polydimethylsiloxane (PDMS) ball were dropped from a height of 1 m, with corresponding infrared photos with temperature scale bars taken at maximum rebound heights at −20°, 20°, and 60°C (Fig. 4B). At −20°C, near the peak loss factor for APFGs, the rebound ratio dropped to as low as 1.25%, indicating more than 98% potential energy dissipation. Across the temperature range from −40° to 80°C, APFGs consistently exhibited much lower rebound ratios compared to PDMS (Fig. 4C). Given their exceptional energy dissipation performance, APFGs are also applicable in protecting fragile objects. For example, in fig. S15, an egg dropped from a height of 1 m broke easily, while a 5-mm–thick damping APFG prevented damage, maintaining the egg’s integrity under both low and high temperatures (movie S2).

To quantify the vibrational damping capabilities of APFGs, we built up an experimental setup, as shown in fig. S16, comprising a 5-mm-thick APFG constrained between a vibrator and a force sensor. With the APFG damper in place, vibrational forces were notably reduced from about 55 to 40% of the control results observed without damping at 10, 20, and 50 Hz (fig. S17). The damper demonstrated substantial reductions in amplitude at wide temperatures, and reduction ratios reached as high as 72, 61, and 52% at −20°, 20°, and 60°C, respectively (Fig. 4D and fig. S18). We further explored the behavior of sand grains on damped versus nondamped surfaces under force vibrations at 50 Hz (fig. S19). On the nondamped surface, sand grains vibrated violently and vanished in 5 s, while those on the damped surface remained stable (Fig. 4E and movie S3). This stability was maintained even at extreme temperatures (−20° and 60°C) (Fig. 4E). Overall, APFGs demonstrated optimal damping properties at the glass transition region.

### APFG dampers for electronic protection and human health care applications

**Fig. 5.**
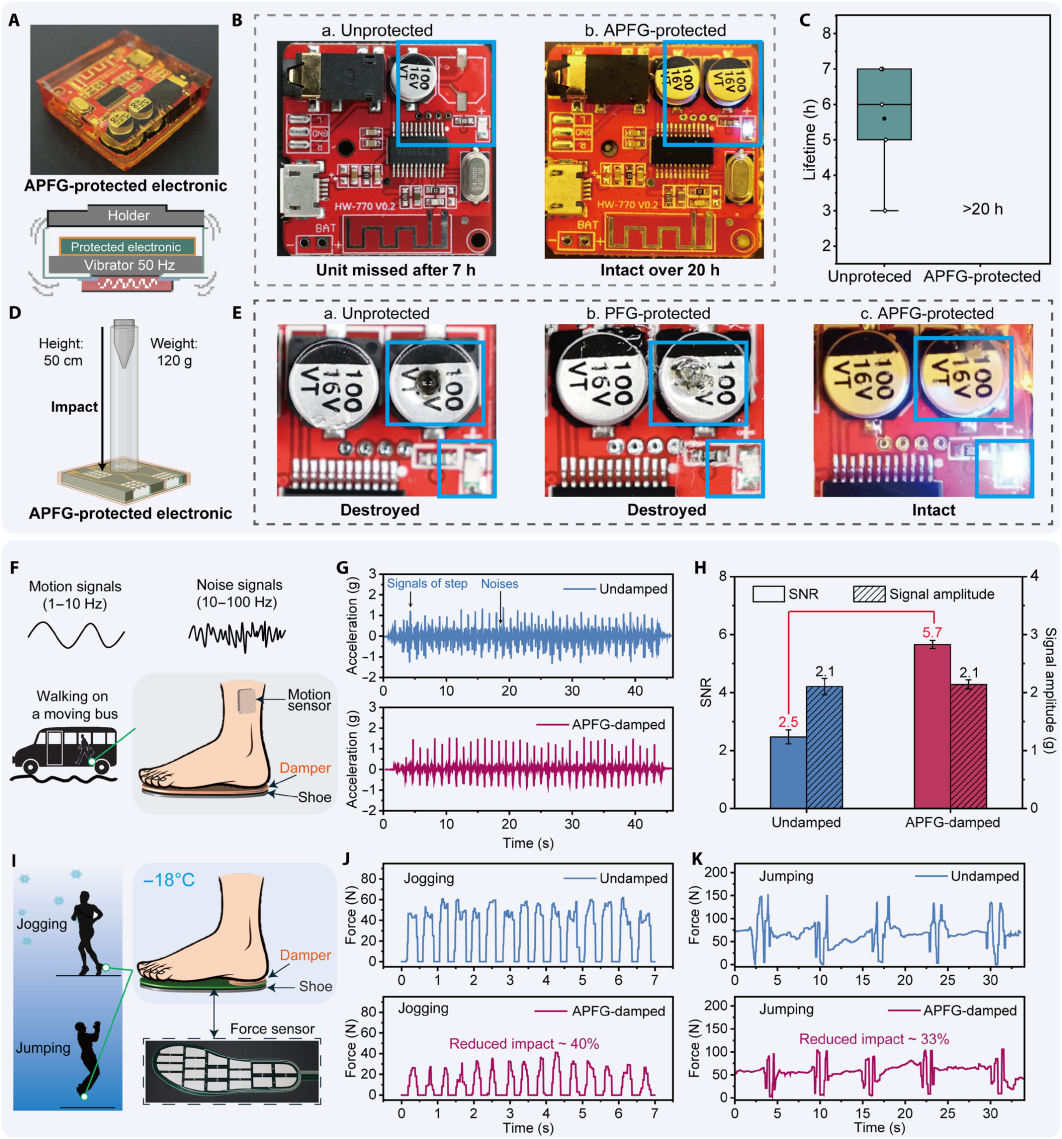
Armored PFG (APFG) dampers for electronic protection and health care applications. (**A**) Photograph of an electric board enclosed within APFG for protection and schematic illustrations of the vibrational damping setup. (**B**) Images of electric boards after prolonged exposure to continuous forced vibrations, comparing unprotected and APFG-protected conditions. (**C**) Lifetime comparison of the electric boards under vibrations with and without APFG protection (*n* = 5 each). (**D**) Schematic demonstration of impact protection, showing an APFG-protected device subjected to a falling plummet. (**E**) Comparative analysis of impact protection performance: unprotected, PFG-protected, and APFG-protected devices. (**F**) Experimental setup for human motion sensing in noisy environments, illustrating motion signals (1 to 10 Hz) and noise signals (10 to 100 Hz) during walking on a bus. The configuration for APFG-damped scenarios is shown. (**G**) Acceleration data during walking on a moving bus, showing the reduction in noise and improved signal clarity with APFG damping. (**H**) Comparison of SNR and the signal amplitude between undamped and damped motion sensing (*n* = 5 each). (**I**) Human motions at low temperatures (−18°C) with an APFG-damped configuration for articulation protection. (**J**) Force signals recorded during jogging, showing a ~40% reduction in impact with APFG dampers. (**K**) Force signals recorded during vertical jumping, demonstrating a ~33% reduction in impact with APFG dampers.

APFG dampers effectively suppress vibrations that could jeopardize the structural stability of sensitive devices. These damping materials are crucial for protecting sophisticated and delicate devices, such as electrical units in cellphones, where various mechanical inputs could shorten their lifespan or cause permanent damage. For example, solder joints on electric boards are sensitive to continuous vibrations, which can gradually degrade the bonding. We visualized the APFG’s damping ability by protecting an electric board subjected to large-amplitude forced vibrations. As shown in Fig. 5A, we sealed the board within the APFG to keep it protected and used the setup to generate stable vibrations, which included a vibrator and a holder. Vertical vibrations were applied continuously at 50 Hz, and the device was evaluated at 1-hour intervals against an unprotected board. Under continuous vibrations and forced contact with the clamp for 7 hours, solder joints of the capacitor on the unprotected board were destroyed, rendering the device nonfunctional. In contrast, the protected board operated normally after 20 hours of exposure to vibrations (Fig. 5B). Among five unprotected sample boards, lifetimes ranged from 3 to 7 hours, whereas no damage was observed on the protected boards after 20 hours (Fig. 5C).

In addition to continuous vibration forces, transient impacts also pose a notable risk to materials and structures, potentially causing immediate damage because of their high magnitude. Fortunately, APFG dampers, with their combination of high damping and good impact tolerance, are suitable for transient impact protection. The APFG damper effectively mitigated impacts, decaying forces within several deformation cycles. As depicted in fig. S20A, the impact force of a falling glass ball was recorded with and without the damper’s protection using a force sensor. Notably, the APFG damper efficiently absorbed transient impacts, registering only the peak impact force once, while the impact continued to propagate with no-damping or low-damping PDMS (fig. S20B). Specifically, the APFG damper reduced the initial impact forces to around 50% of the original values at first impact, and more than 80% amplitude was eliminated during the second complete cycle across different falling heights. In comparison, PDMS maintained more than 85% peak forces (fig. S20C). Moreover, the armor provides high strength, preventing permanent damage to materials. Conventional damping materials like PFGs may reduce impacts but are prone to failure. By combining excellent damping with enhanced puncture and indentation resistance, APFGs represent an ideal solution for transient impact protection of delicate devices. We conducted three controlled experiments under similar conditions, applying impacts by releasing a 120-g plummet from a height of 50 cm onto three electric boards to simulate impact protection (Fig. 5D). One was placed on a table (without protection), while the others were embedded in a PFG damper (damping but impact sensitive) or an APFG damper (damping and impact resistant) (Fig. 5E). Following impacts exerted by the plummet on the capacitor locations, circuits without protection or those protected by the PFG damper were destroyed, and the PFG was punctured. However, the device embedded in the APFG damper remained intact, with no visible damage to the capacitor, highlighting the superior impact protection performance of APFG materials (Fig. 5E and movie S4).

The broad-temperature damping and impact resistance properties of APFGs make them ideal candidates for applications in motion sensing and health care, particularly in human motions. Motion sensing is of great importance in health care, robotics, and human-machine interaction by converting human movements into recognizable digital signals. However, external environmental vibrations, such as those encountered when riding a bike over a bumpy road or walking on a moving bus, often interfere with these signals, leading to poor signal quality and complicating data analysis (*10*). Human motion signals typically have low frequencies (1 to 10 Hz), while noise signals tend to be at higher frequencies (10 to 100 Hz). Fortunately, APFGs, with their ability to effectively dampen high-frequency noise, offer an important advantage in improving signal clarity in noisy environments (Figs. 3A and 5F).

To demonstrate this, we embedded the APFG damper in a shoe pad and compared it to an undamped control. Motion signals were collected by a sensor as the experimenter walked on a stable treadmill and a moving bus (Fig. 5F). On the treadmill, where vibrations were minimal, the motion signals were clear and similar for both the damped and undamped conditions (fig. S21). However, when walking on a moving bus, the undamped signals showed noticeable noises, making it difficult to distinguish the motion signal. In contrast, the damped signals clearly suppressed the noise (Fig. 5G). The signal-to-noise ratio (SNR) was calculated to quantify this improvement. The undamped signals had a low SNR of 2.5, which was increased to 5.7 with the APFG damper without compromising the signal amplitude, highlighting its potential for enhancing motion sensing in noisy environments (Fig. 5H).

In addition to noise reduction, APFGs also provide consistent damping and impact resistance, even at low temperatures. Traditional shoe soles lose their damping and impact protection capabilities in cold environments, compromising both comfort and safety (Fig. 1A). To evaluate the performance of APFGs under these conditions, we conducted experiments at −18°C, recording impact forces during jogging and jumping motions using a force sensor (Fig. 5I). The APFG dampers effectively reduced peak impact forces by ~40% during jogging and 33% during jumping, demonstrating their superior impact protection compared to undamped controls (Fig. 5, J and K). These results highlight the potential of APFGs in providing durable damping and protection for health care applications, particularly under low-temperature conditions.

## DISCUSSION

In conclusion, we have developed an innovative strategy for creating protective materials that overcome the traditional damping-modulus trade-off, achieving exceptional damping and impact protection across a wide temperature range. As an example, a high-performance protective material was facilely prepared by copolymerizing AA and HEMA monomers in the presence of PEG fluid, followed by surface cross-linking with Fe^3+^ solutions to create an armor-core structure. The properties of obtained APFGs can be finely tailored by tuning the composition of the gels and modifying the posttreatment conditions. The materials, working as dampers, can be readily applied for damping and protection. The APFG dampers help to extend device lifetimes under continuous vibration and resist external impacts to ensure durable functionality. In human motion applications, APFGs enhance motion sensing and articulation protection in noisy and low-temperature environments, improving SNRs and reducing peak impact forces during different motions.

Our strategy provides pathways for designing materials that integrate conventionally irreconcilable properties into anisotropic yet continuous materials. Such a structural design can be further exploited in various ways. For example, the treatment is adaptive to complex topologies and structures, demonstrating superior efficiency compared to bottom-up methods. Specifically for damping materials, the low integrity and complicated fabrication of constrained damping systems can be addressed through one-step treatment, applicable to those unconstrained, programmed, or metastructures in the future work. This strategy also exemplifies achieving uncompromised properties in a single material, effectively resolving dissipation-modulus, cross-linking-damping, and strength-deformability trade-offs.

## MATERIALS AND METHODS

### Materials

Iron(III) chloride hexahydrate (FeCl_3_·6H_2_O) and PEG with different average molecular weights were purchased from Aladdin. AA, HEMA, 2-hydroxy-2-methyl propiophenone, and phenylbis(2,4,6-trimethylbenzoyl)phosphine oxide were purchased from Energy Chemical. All these materials were used as received.

### Preparation of PFGs, APFGs, and protective dampers

To prepare PFGs, for example, PFG-400, typically 1 g of AA, 1 g of HEMA, 2 g of PEG 400, and 0.02 g of 2-hydroxy-2-methyl propiophenone (1 wt % to the monomer weight) were vortexed for 2 min to obtain thoroughly mixed precursor solution. After 1 min of sonication to remove bubbles, the precursor was injected into a silicone spacer sandwiched by two PET films and glass laps. UV irradiation (256 nm) was applied for 30 min to obtain the polymerized material, which was cut into specific shapes for further characterization. To prepare APFGs, first, FeCl_3_·6H_2_O was dissolved in PEG 400 to prepare the solution with specific Fe^3+^ molar concentrations (0.5, 1, and 2 M). The above-obtained PFGs with preset shapes were immersed in the solution for certain time to prepare core-armor gels. To embed the electric board in the APFG damper, the board (6-mm height) was fixed in a silicone mold (10-mm height) and sandwiched by release films, followed by injection of the precursor, in situ UV curing, and Fe^3+^ treatment. The impact was targeted at one specific capacitor on the circuit, and the thickness of the protective APFG layer is 4 mm. The board has Bluetooth audio decoding functionality and light indicator, which were used to validate its intactness.

### Mechanical tests

Tensile, compression, lap shear, puncture, and tear tests were conducted on a universal testing machine (UTM 68TM-5, Instron). Dumbbell-shaped specimens (20 mm in gauge length, 4 mm wide, and 1 mm thick) were used for tensile tests, with a 100-N load cell and a strain rate of 5 min^−1^. Cylindrical specimens (15 mm in diameter and 5 mm thick) were used for compressive tests with a 5-kN load cell and a strain rate of 1 min^−1^. Puncture tests followed an ASTM F1306 standard, and tear tests followed an ASTM D624 standard with a 100-N load cell.

### DMA tests and TTS analysis

DMA tests were conducted on a dynamic mechanical analyzer (DMA850, TA Instruments). Typically, a strain sweep test was first applied to each specimen to determine the linear region. The oscillation strain was raised from 0.01 to 2% with a frequency fixed at 1 Hz at RT. Then, frequency sweep tests were conducted from 0.1 to 150 Hz with a fixed strain (usually 0.1%) within the linear region. In a temperature ramp test, the temperature was ramped at 3°C·min^−1^ with the strain and frequency fixed at 0.1% and 1 Hz, respectively. Rectangular specimens with dimensions of 8 mm by 1 mm and a gauge length of 10 mm were used in tensile measurements, while cylindrical specimens with a 3-mm diameter and 5-mm thickness were used for compressive measurements. TTS analysis was performed on the basis of the results of a temperature-dependent multifrequency sweep. The sweep interval was 0.1 to 10 Hz. The horizontal shift factors were calculated by the Williams-Landel-Ferry equation. The activation energy *E*_a_ was calculated on the basis of the Arrhenius equation *a*_*T*_ = *A*exp(−*E*_a_/*RT*), where *a*_*T*_ is the horizontal shift factor, *A* is a constant, *R* is the gas constant, and *T* is the temperature.

### Characterizations

Cross-sectional pictures were taken on an optical microscope (BX53M, Olympus) with 10× enlargement. The thickness of the cross-linked layer was directly calculated from the images. SEM cross-sectional images were obtained by a FIB-SEM dual system (TESCAN, SOLARIS GHM) at a voltage of 5 kV. A conductive Pt layer of 6 nm in thickness was coated on the materials before imaging. Energy-dispersive x-ray spectroscopy mapping was carried out with respect to Fe, C, and O elements. XPS measurements were performed using a spectroscope (AXIS SUPRA+, Kratos), and survey, C 1s, O 1s, and Fe 2p spectra were obtained. Infrared spectra were recorded on an FTIR spectrometer (Spectrum Two, PerkinElmer) from 400 to 4000 cm^−1^. Transmittance spectra were obtained from a UV-vis spectrometer (UV1900, Shimadzu). The specimens used have a pristine thickness of 1 mm. The *T*_g_ of the materials was obtained from a DSC test by a calorimeter (DSC2500, TA Instruments). The sample was heated to 100°C then cooled to −80°C at a rate of 10°C·min^−1^ for two cycles, and the sample mass was 10 mg. Durometer hardness tests were carried out according to the ASTM D2240 method by a type-A Shore durometer (LX-A, Sanliang), where samples have a thickness of 5 mm. Nanoindentation tests were conducted on a nanoindenter (Hysitron TI 980 TriboIndenter, Bruker). The load was ramped to 100 μN at a rate of 10 μN·s^−1^, held for 20 s, and then ramped to 0. The hardness was calculated by the Oliver-Pharr indentation method, which is defined as the maximum load over the contact area.
