# Supplementary material for: Armored polymer-fluid gels with integrated damping and impact protection across broad temperatures
Source: Sci Adv. 2025 Apr 9;11(15):eadv5292. doi: 10.1126/sciadv.adv5292 (PMC11980849; doi:10.1126/sciadv.adv5292)
Supplement: Supplementary file 1 — Figs. S1 to S21 Tables S1 and S2 Legends for movies S1 to S4 [file sciadv.adv5292_sm.pdf]

Science Advances
AAAS

Supplementary Materials for

Armored polymer-fluid gels with integrated damping and impact protection across broad temperatures

Guoqing Chen *et al.*

Corresponding author: Zhenwu Wang, wangzhenwuup@163.com; Qi Zhang, qizhang@cuhk.edu.cn

Sci. Adv. **11**, eadv5292 (2025)
DOI: 10.1126/sciadv.adv5292

The PDF file includes:

Figs. S1 to S21
Tables S1 and S2
Legends for movies S1 to S4

Other Supplementary Material for this manuscript includes the following:

Movies S1 to S4

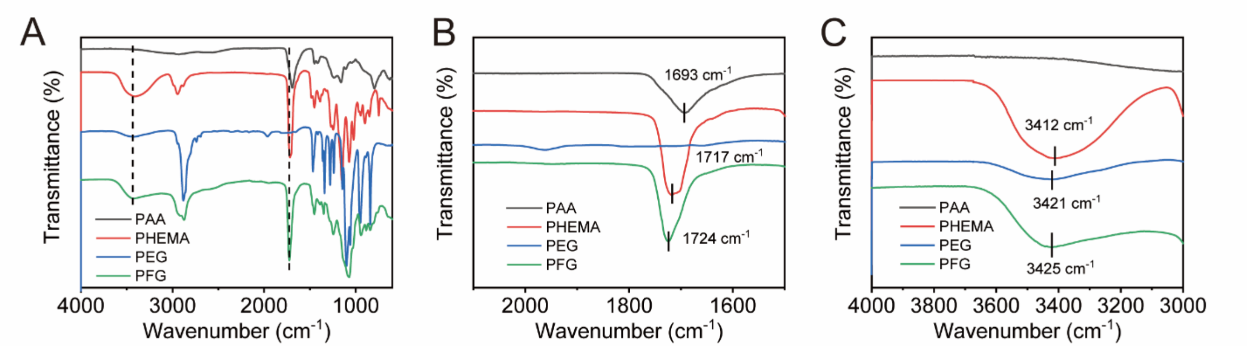

Fig. S1.

Analysis of FTIR spectra of PFGs. (**A**) ATR-FTIR spectra of PAA, PHEMA, PEG and PFG. (**B**) Shifts of characteristic peaks of carboxyl groups. (**C**) Shifts of characteristic peaks of -OH groups.

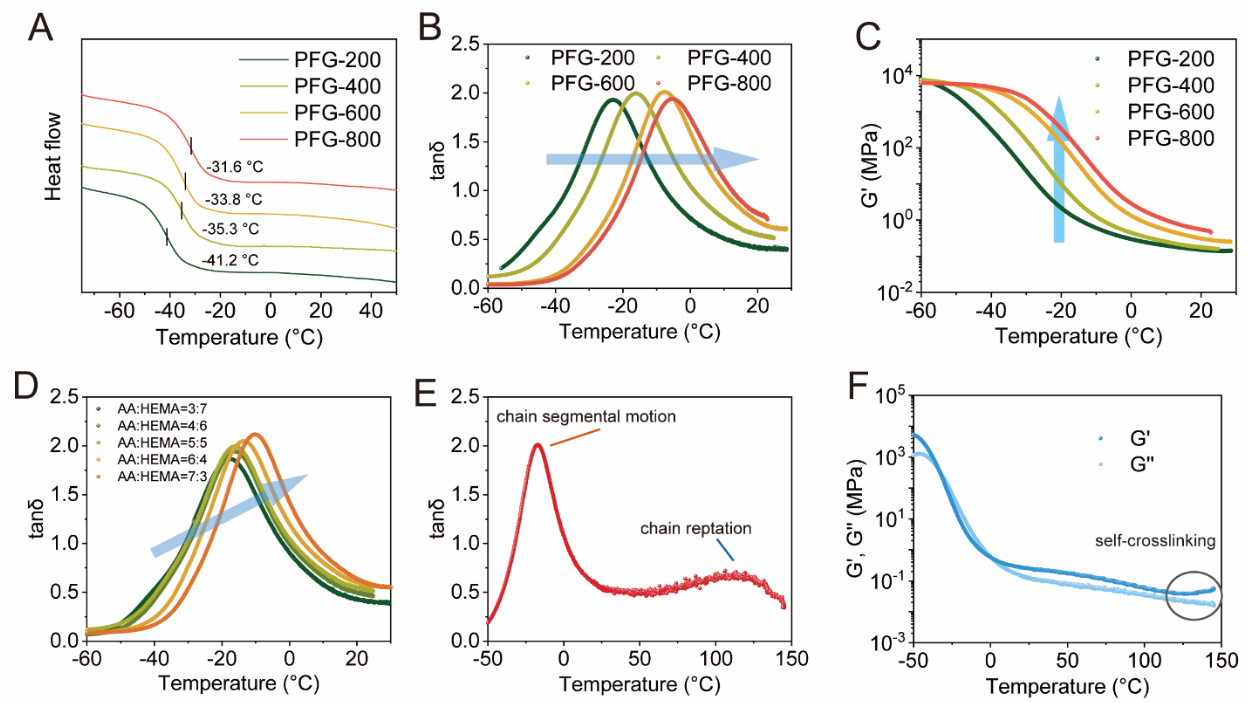

Fig. S2.

Regulations on the recipe of the PFG regarding the damping performance. (**A**) DSC traces and glass transition temperatures of PFGs with PEG solvent of different molecular weights. (**B**) Loss factor curves of PFGs from low temperature to room temperature and the dependence on PEG's molecular weight. (**C**) Storage moduli of PFGs from low temperature to room temperature and the dependence on PEG's molecular weight. (**D**) Loss factor curves of PFGs with different monomer weight ratios from low temperature to room temperature. (**E**) The temperature-dependent loss factor curve of PFG-400 and the characteristic relaxation frequencies corresponding to polymer network and linear PEG. (**F**) The temperature-dependent storage and loss modulus curves of PFG-400.

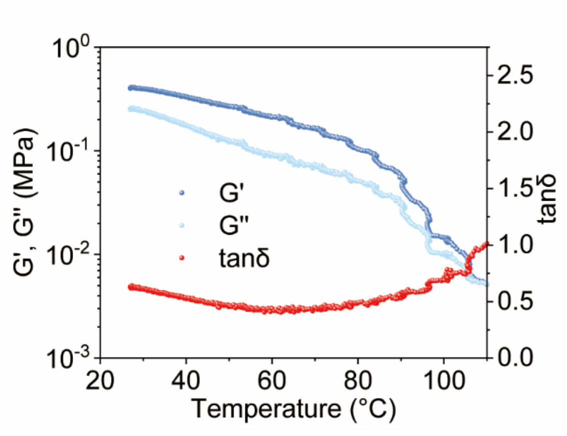

Fig. S3.

Temperature-dependent DMA test results of PFG-400 gel with only AA as the monomer.
The moduli decreased substantially at elevated temperatures.

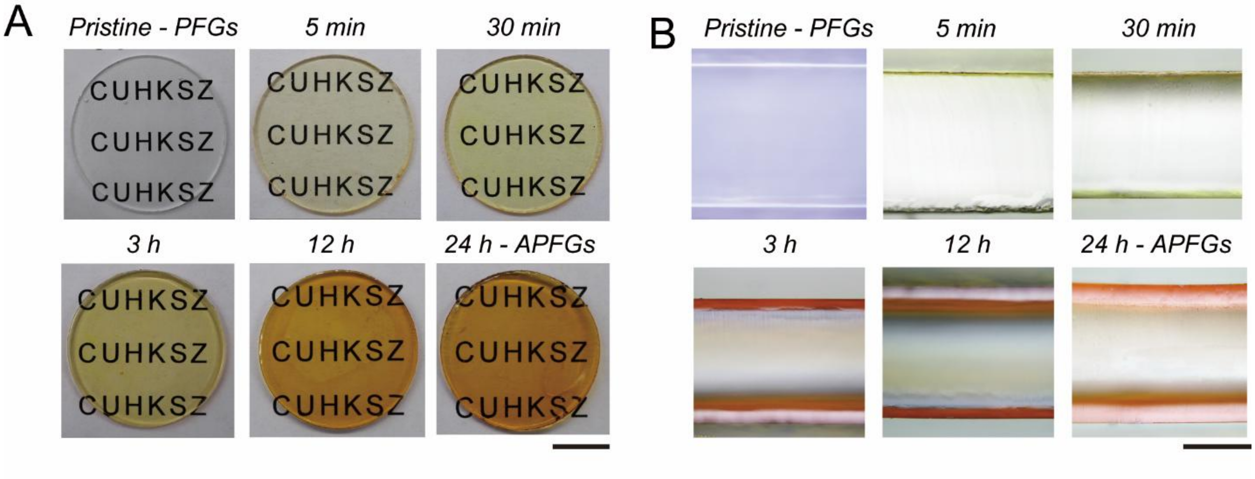

Fig. S4

Optical images of APFGs with different soaking times. (**A**) Surface images (scale bar: 1 cm) and (**B**) Cross-sectional images (scale bar: 200 μm) of pristine LPGs and specimens after certain soaking time.

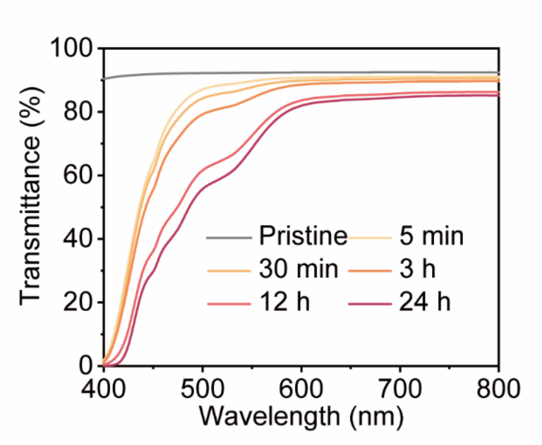

Fig. S5.

UV-vis spectra of pristine APFGs and specimens after certain soaking time. Transparency decreases as the soaking time of APFGs increases,

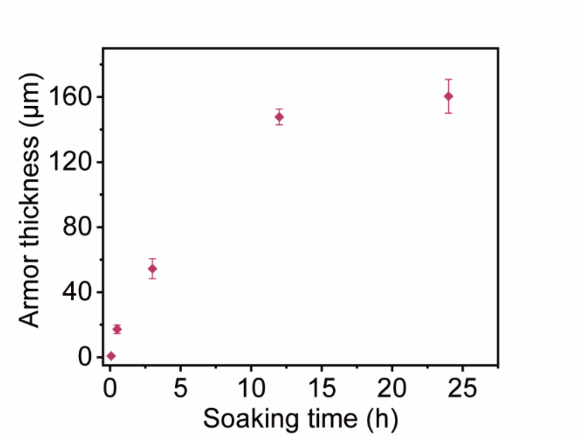

Fig. S6.

Crosslinked armor thickness of APFG specimens with varied soaking time. The armor thickness gradually increases with increased soaking time.

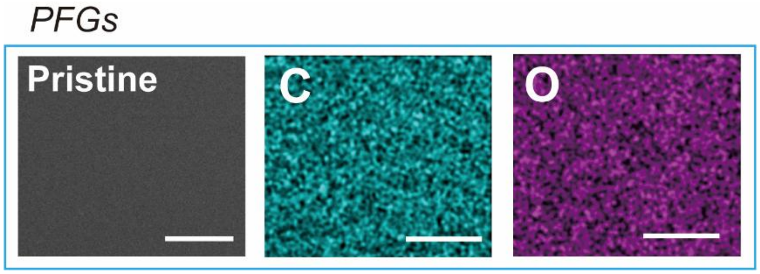

Fig. S7.

The SEM image of PFGs and the corresponding EDS mapping of C, O and Fe. PFGs demonstrate homogeneous structures (scale bar: 50 μm).

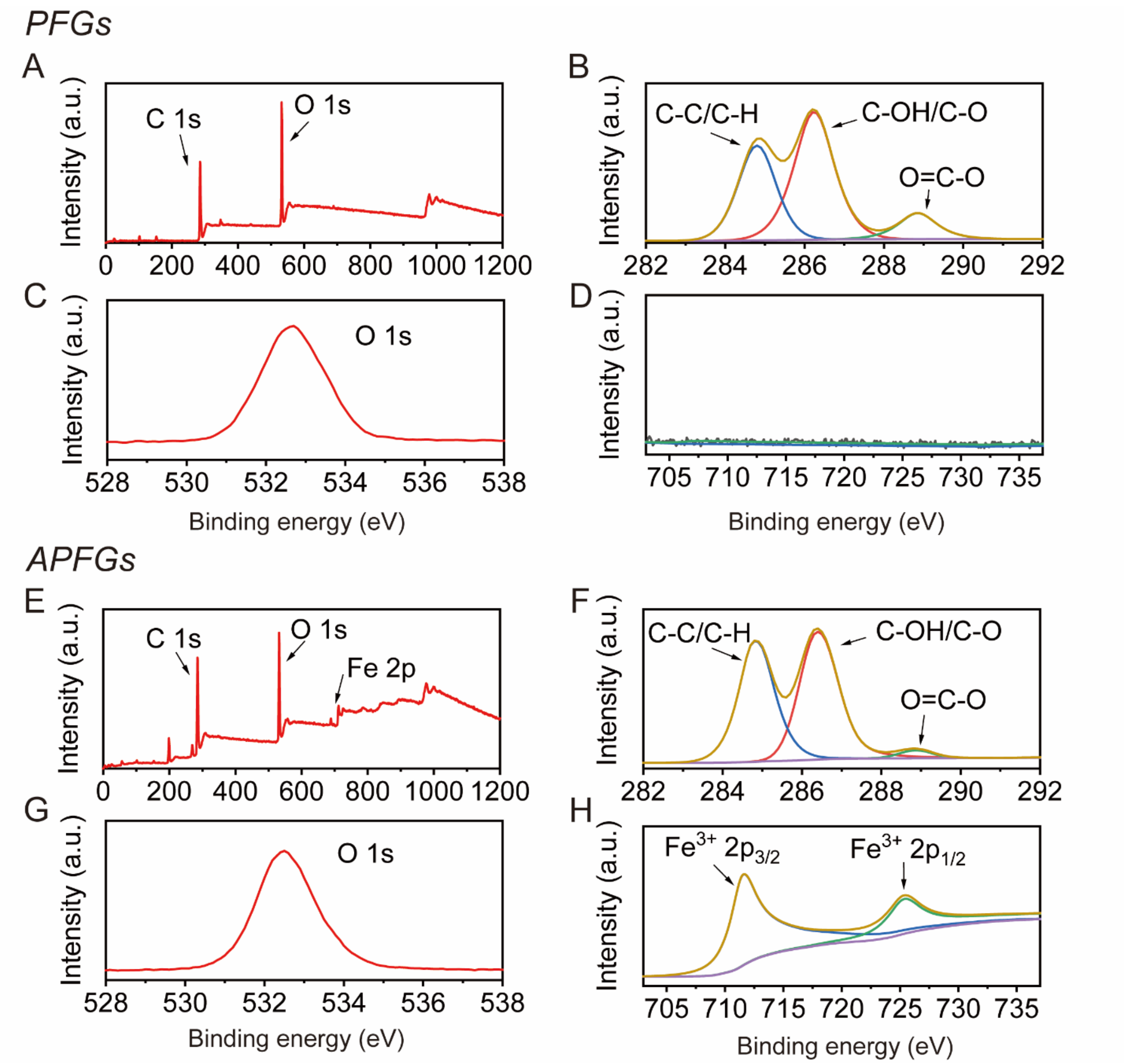

Fig. S8.

XPS spectra of PFGs. (**A**) Survey, (**B**) C 1s, (**C**) O 1s, (**D**) Fe 2p. XPS spectra of APFGs: (**E**) Survey, (**F**) C 1s, (**G**) O 1s, (**H**) Fe 2p.

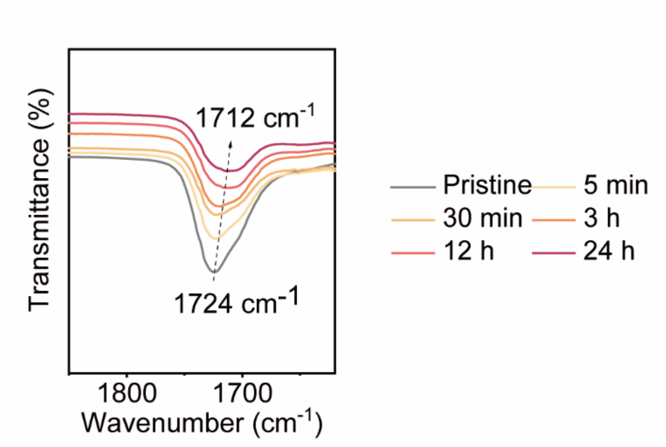

Fig. S9.

FTIR spectra of pristine PFGs and APFG specimens after certain soaking time. Corresponding characteristics peak of carbonyl groups demonstrates obvious shifts during soaking treatment.

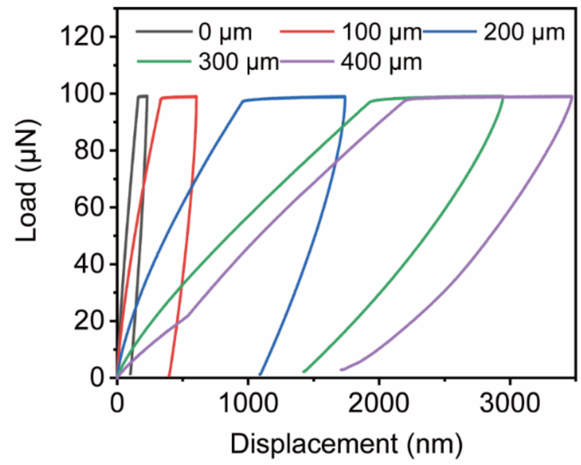

Fig. S10.

Load-displacement curves of nanoindentation tests of APFGs. Results are obtained at different cross-sectional distances from the outmost armor.

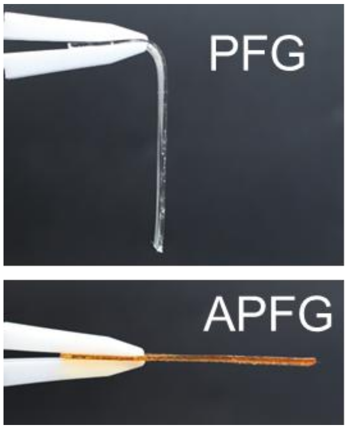

Fig. S11.
Shape-retention ability of PFG and APFG under gravity.

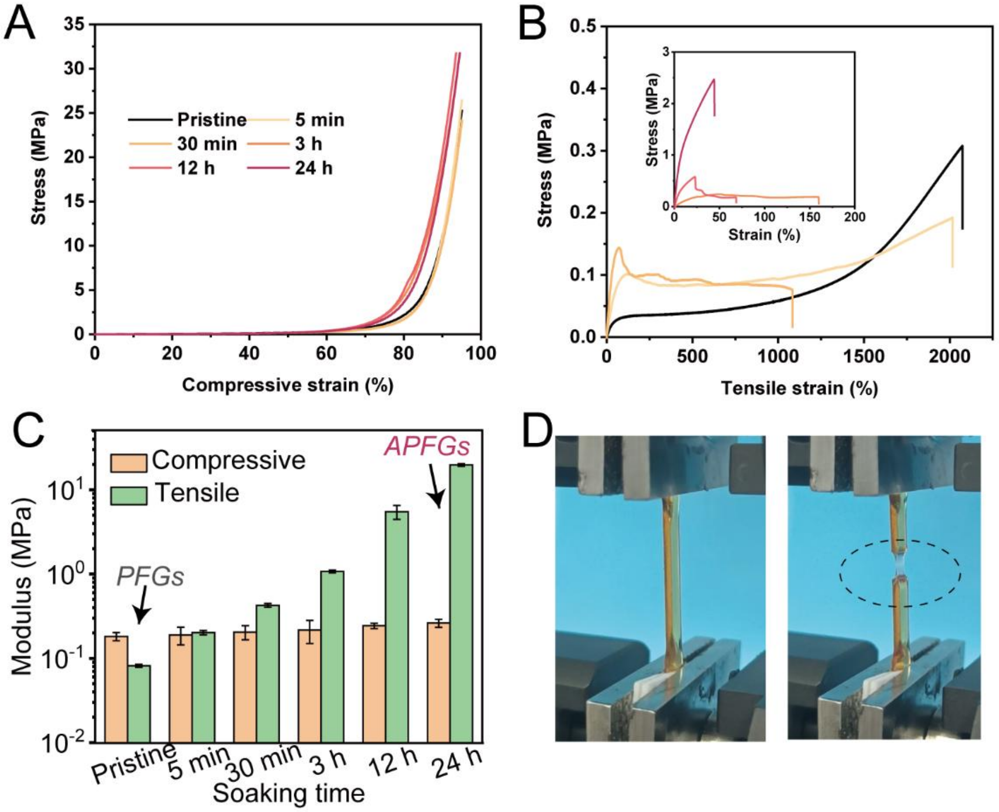

Fig. S12.

Compressive and tensile tests results of APFGs with different soaking times. (**A**) Compressive and (**B**) tensile stress-strain curves of pristine and specimens with different soaking time. (**C**) Compressive and tensile moduli with respect to soaking time. (**D**) Yielding and necking observed during tensile tests of specimen treated for 3 h.

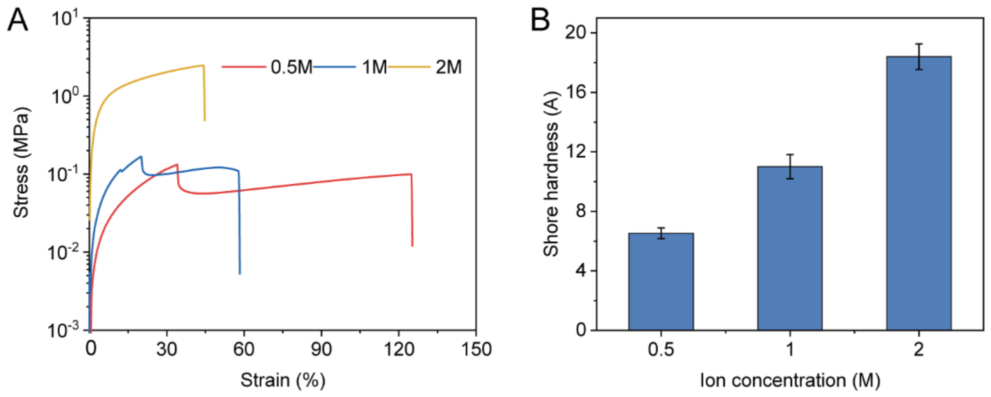

Fig. S13.

Results from tensile and Shore hardness tests of APFGs soaked in ion solutions with varied concentrations. (**A**) Tensile stress-strain curves of specimens with 24 h soaking time in solutions with varied Fe^{3+} concentration. (**B**) Shore hardness of the corresponding specimens.

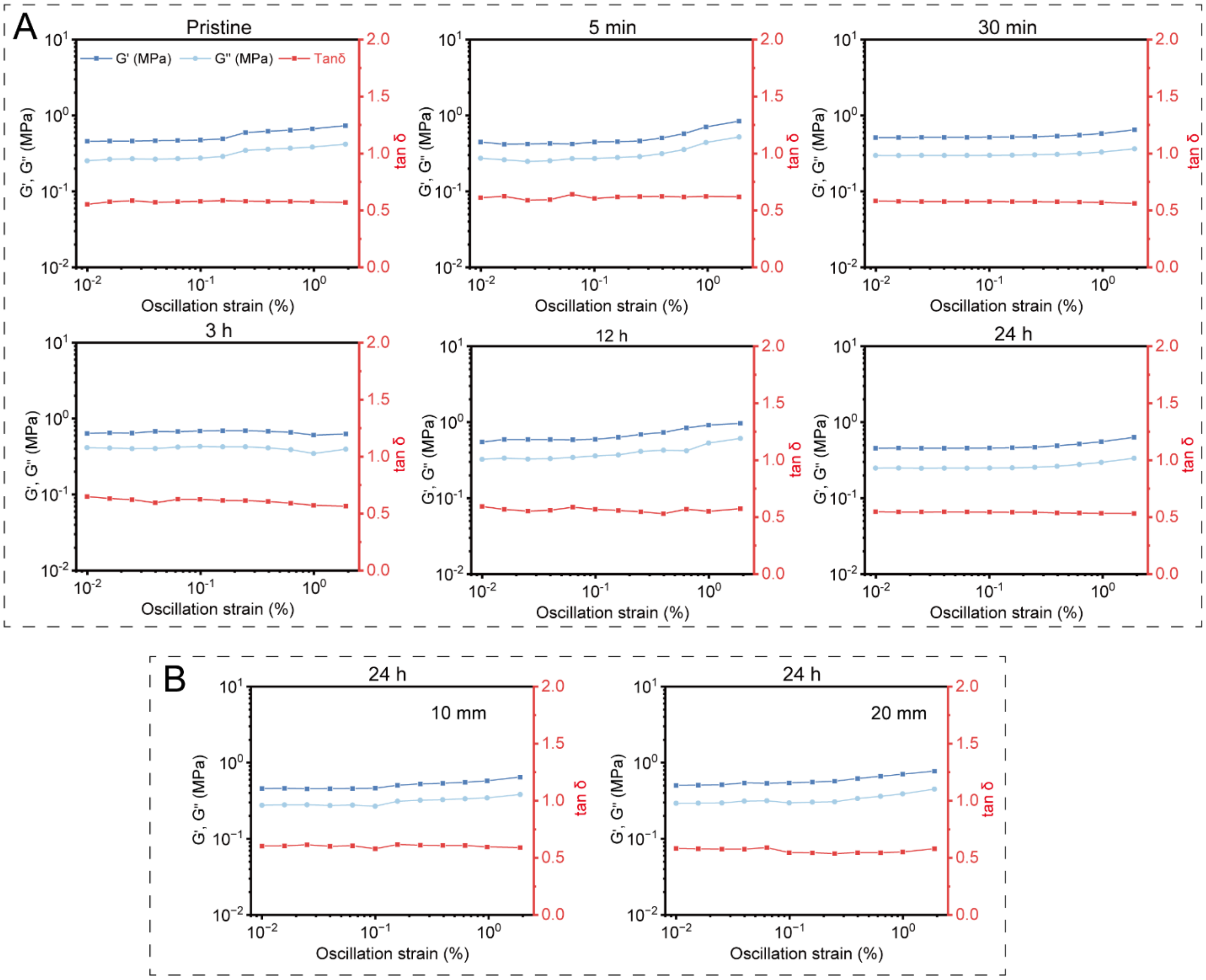

Fig. S14.

Strain sweep compressive DMA test results of APFGs. Results regarding (**A**) different soaking time. (**B**) varied sample thickness.

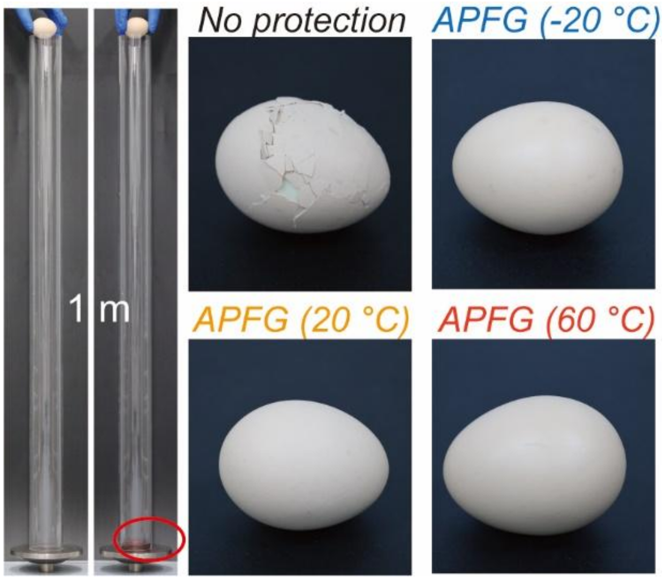

Fig. S15.

Protection of falling egg at varied temperatures by APFG dampers. Eggs are well protected at different temperatures.

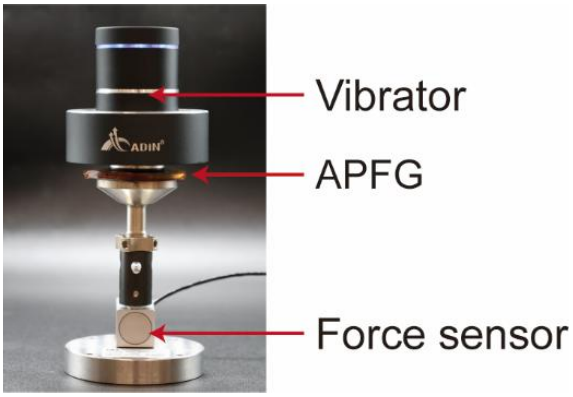

Fig. S16.
Experimental setups for vibrational force sensing damped by APFG.

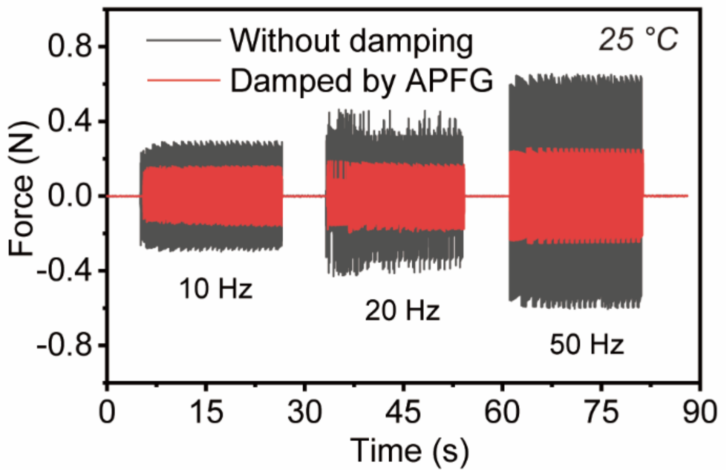

Fig. S17.

Vibrational damping performance of APFG dampers at different frequencies. APFGs dampers demonstrate effective vibrational damping at different frequencies.

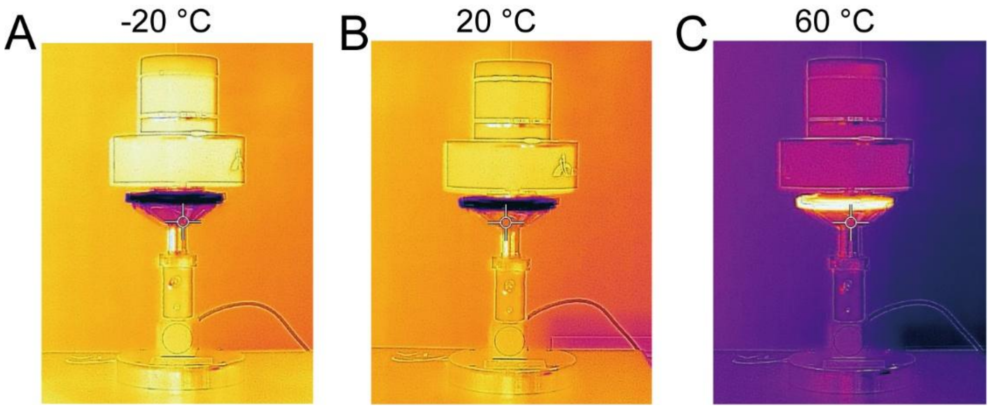

Fig. S18.

IR photos of the experimental setup at different temperatures. (**A**) -20 °C, (**B**) 20 °C, and (**C**) 60 °C.

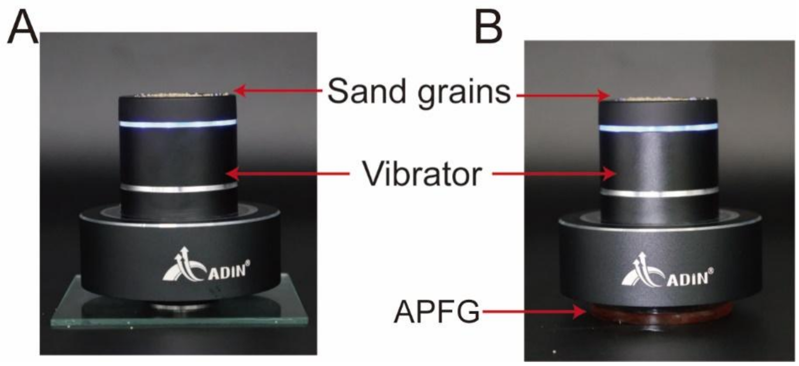

Fig. S19.

Experimental setups for sand motion on vibrating surfaces. (**A**) Vibrations without damping and (**B**) damped by APFG.

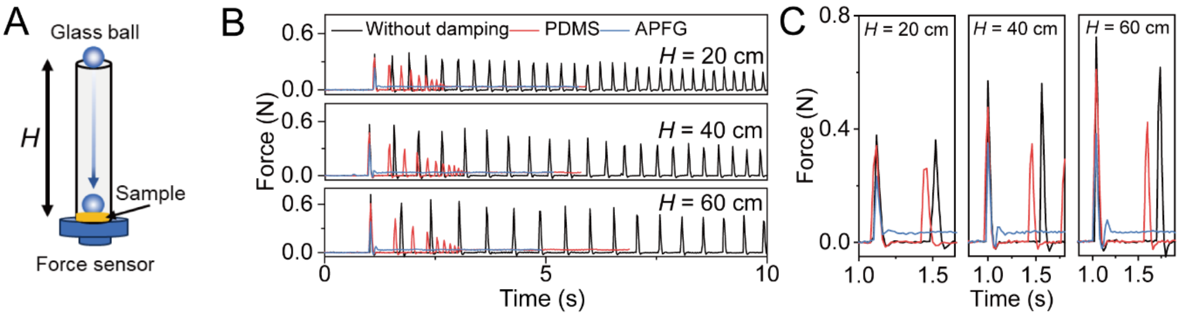

Fig. S20.

Evaluation of impact force reduction performance by APFG dampers. (**A**) Setup for detecting impact force of a glass ball released from certain height. (**B**) Curves of the detected forces when the ball hits the force sensor from different release heights without damping, with PDMS or with APFG. (**C**) Differences in the detected peak forces and decay rates in the first two hits of the three testing conditions.

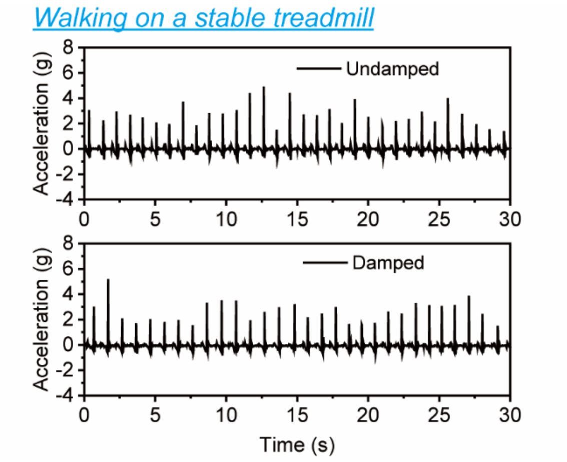

Fig. S21.

Acceleration signals of walking on a stable treadmill in damped and undamped situations. The results are similar in the two cases, indicating little influence on original signals while removing noises.

Table S1.

Comparison of highly damping temperatures and tensile modulus of reported materials.

	Materials	Lowest highly damping T (°C)	Highest highly damping T (°C)	Effective T-range (°C)	Modulus (MPa)	Journal name and year	Ref No.
Gel	**APFG**	-45	135	**180**	**20.04**	**This work**	/
	Polymer fluid gel	-50	120	170	~0.01	Nat. Commun. (2021)	18
	Organogel	-10	60	70	~0.003	Adv. Mater. (2023)	39
	Polyborosiloxane gel	60	150	180	~0.05	Polymers (2020)	40
	Mineral oil gel	50	150	100	~0.04	Smart Mater. Struct. (2016)	41
Elastomer	Liquid crystal elastomer	-10	50	60	~1	Nat. Commun. (2021)	33
	PDMS elastomer	-30	150	180	~0.05	Chem. Mater. (2004)	34
	Chlorobutyl rubber	-60	20	80	~0.7	Polym. Test. (2019)	35
	Superamolecular elastomer	-25	40	65	~0.5	ACS Appl. Mater. Interfaces (2022)	36
	Polyurethane elastomer	0	35	35	~0.25	Euro. Polym. J. (2021)	37
	Fluorinated polymer	0	60	60	~1	Adv. Mater. (2023)	38
	Liquid crystal semi-IPN	48	116	68	~16	Nat. Commun. (2024)	39
Composite	Elastomer composite	-51	125	176	~0.0076	Chem. Eng. J. (2023)	19
	Polyurethane composite	20	50	30	~12	Compos. B Eng. (2022)	42

Table S2.

Comparison of highly damping temperatures and tensile modulus of reported materials.

Material	Strain (%)	Stress (MPa)	Reference	No.
**APFG**	**95**	**32**	**This work**	/
PBA hydrogel	~65	< 0.05	*Nat. Commun.* 2021, 12, 3610.	18
RCP	< 80	~ 2	*Biomacromolecules* 2014, 15(9), 3358-3365.	51
PVA/(PVA-MA)-*g*-PNIPAM hydrogel	< 90	3.8	*ACS Appl. Mater. Interfaces* 2021, *13*(11), 12689-12697.	52
BC-PHEMA	47	2	*Compos. B Eng.* 2015, 76, 292-299.	53
BC-PVA	< 45	23	*Adv. Funct. Mater.* 2020, *30*(36), 2003451.	54
DN2.5+EG	80	3.5	*J. Mater. Chem. A.* 2020, 8, 6219-6228.	55

Movie S1.
Demonstrations of mechanical properties of PFG and APFG.

Movie S2.
Protection of a falling egg by APFG damper.

Movie S3.
Visualization of vibrational damping ability of APFG dampers by sand vibration tests.

Movie S4.
Impact protection of electronics of by the APFG.
